# 

**Published:** 1904-12

**Authors:** 


### BOOKS RECEIVED.

A Textbook of Practical Therapeutics, with especial reference to the application of Remedial Measures to Disease and their Employment upon a Rational Basis. By Hobart Amory Hare, M.D., B.Sc., Professor of Therapeutics and Materia Medica in the Jefferson Medical College, Philadelphia. With special chapters by G. E. de Schweinitz, Edward Martin and Barton C. Hirst. Tenth edition, enlarged, thoroughly revised and largely rewritten. Octavo, 908 pages, with 113 engravings and 4 full-page colored plates. Philadelphia and New York: Lea Brothers & Company. 1904. (Price: cloth, $4.00 net; leather, $5.00; half morocco, $5.50 net prices.)

A Treatise on Bright's Disease and Diabetes, with especial reference to Pathology and Therapeutics. By James Tyson, M.D., Professor of Medicine in the University of Pennsylvania. Second edition, illustrated. Octavo, 381 pages. Including a section on the Ocular Changes in Bright's Disease and in Diabetes, by G. E. de Schweinitz. Philadelphia: P. Blakiston's Son & Company. 1904. (Price, $4.00.)

Blakiston's Quiz Compends. A Compend of Medical Latin. By W. T. Saint Clair, A.M., Professor of the Latin Language and Literature in the Male High School of Louisville, Ky. Duodecimo, 131 pages. Second edition, revised. Philadelphia: P. Blakiston's Son & Company. 1904. (Price, $1.00.)

Appendicitis and Other Diseases about the Appendix. By Bayard Holmes, B.S., M.D., Professor of Surgery in the University of Illinois, Chicago. Three hundred and fifty pages. New York: D. Appleton & Company. 1904.

Twenty-third Annual Report of the State Department of Health of New York. For the year ending December 31, 1902. With maps. Daniel Lewis, M.D., Commissioner.

